# Tubular basement membrane deposits after allogeneic hematopoietic stem cell transplantation

**DOI:** 10.1186/s12882-023-03296-x

**Published:** 2023-08-18

**Authors:** Wenyan Zhou, Chaojun Qi, Minfang Zhang, Xiaotao Hou, Zhaohui Ni

**Affiliations:** 1 Department of Nephrology, Molecular Cell Lab for Kidney Disease, Shanghai Peritoneal Dialysis Research Center, Uremia Diagnosis and Treatment Center, Ren Ji Hospital, Shanghai Jiao Tong University School of Medicine, 160 Pujian Road, Shanghai, 200127 China; 2 Guangzhou KingMed Center for Clinical Laboratory, Guangzhou International Biotech Island, Building 3 Standard Property Unit 3, No.10 Luoxuan 3Rd Road, Guangdong, 510005 China

**Keywords:** Tubular basement membrane deposits, Hematopoietic cell transplantation, Allogeneic

## Abstract

**Background:**

Extraglomerular immune complex deposition is rare and only a few membranous nephropathy cases with tubular basement membrane deposits have been reported following allogeneic hematopoietic stem cell transplantation.

**Case presentation:**

We reported a 56-year-old man with increased serum creatinine after allogeneic hematopoietic stem cell transplantation who underwent a renal biopsy. Tubular interstitial nephritis was identified on light microscope. The unique histologic features were diffuse tubular basement membrane immune complex deposition detected by both immunofluorescence and electron microscopy, while the glomerular involvement was inconspicuous. The differential diagnosis from other forms of tubular basement membrane deposition is discussed.

**Conclusion:**

Diffuse granular tubular basement membrane immune complex deposition with minimal glomerular involvement is also a manifestation of renal complication in hematopoietic stem cell transplantation recipient. However, the exact mechanism and target antigen remains unknown.

## Background

Tubular basement membrane (TBM) immune complexes can be seen in underlying autoimmune diseases (e.g. systemic lupus erythematosus, Sjogren syndrome, IgG4-related disease), monoclonal immunoglobulin deposition disease, infections (such as polyomavirus nephropathy), and drug-induced tubulointerstitial nephritis. Other rare etiologies include anti-brush border autoantibody tubulointerstitial nephritis, anti-TBM disease, giant cell tubulitis with TBM deposits, and tubulointerstitial-predominant fibrillary nephritis. Recently, five cases of membranous nephropathy with extensive TBM deposits following allogeneic hematopoietic stem cell transplantation (HSCT) were reported by Nasr et al. [1], and they postulated that the potential pathogenesis involves chronic graft-vs-host disease (cGVHD)-driven antibodies against glomerular and TBM components. To our knowledge this is the first report of diffuse TBM immune-complex deposition with minimal glomerular involvement after allogeneic HSCT.

## Case presentation

A 56-year-old man had a history of acute monocytic leukemia and underwent allogeneic HSCT about 10 years ago. The GVHD prophylaxis was unknown, and immunosuppression was discontinued nearly 1 year after transplantation. He was admitted to our hospital in 2023 due to increased creatinine (184 umol/L), combined with low molecular weight proteinuria. Fanconi syndrome and renal tubular acidosis was not manifested. His comorbidities included hypertension and hyperlipidemia, which were treated with irbesartan and atorvastatin, respectively. He denied diabetes mellitus, recent infection, or a family history of renal disease. During the hospital course, he had no complaints of skin rashes, arthralgia, dry mouth and eyes, chest tightness, edema, or gastrointestinal symptoms. Physical examination revealed normal body temperature (36.4℃), heart rate (73 beats/minute), body mass index (20.8 kg/m^2^), and high blood pressure (150/93 mmHg). No pitting edema was found in his bilateral lower extremities. Laboratory evaluation showed that his serum creatinine was 184.9 umol/L, 24-h urine protein was 0.32 g (the main component was low molecular weight proteinuria) and urine albumin to creatinine ratio was normal. Microscopic hematuria was not detected by urinalysis. Serum albumin level was 44.8 g/L. Whole blood cell analysis showed white blood cell was 6.41 × 10^9^/L (neutrophil 56%, lymphocyte 27%, monocyte 10%, Eosinophils 6%, and basophilic granulocyte 1%), hemoglobin was 119 g/L, and platelet was 149 × 10^9^/L. Extensive laboratory findings, including inflammatory index (C-reactive protein, erythrocyte sedimentation rate, white blood cell, procalcitonin), anti-streptolysin O, virus (polyomavirus, cytomegalovirus, hepatitis B and C, human immunodeficiency virus, adenovirus, respiratory syncytial virus, and herpes simplex virus), tuberculosis, and syphilis were negative. Immunology tests (immunoglobulin levels, serum complement levels, double-stranded DNA, antinuclear antibody, anti-neutrophil cytoplasmic antibody, rheumatoid factor, SSA, SSB), serum and urine immunofixation, serum free light chain ratio, and tumor markers were all within the normal range. Abdominal ultrasound detected normal size and morphology of both kidneys.

**Fig. 1:**
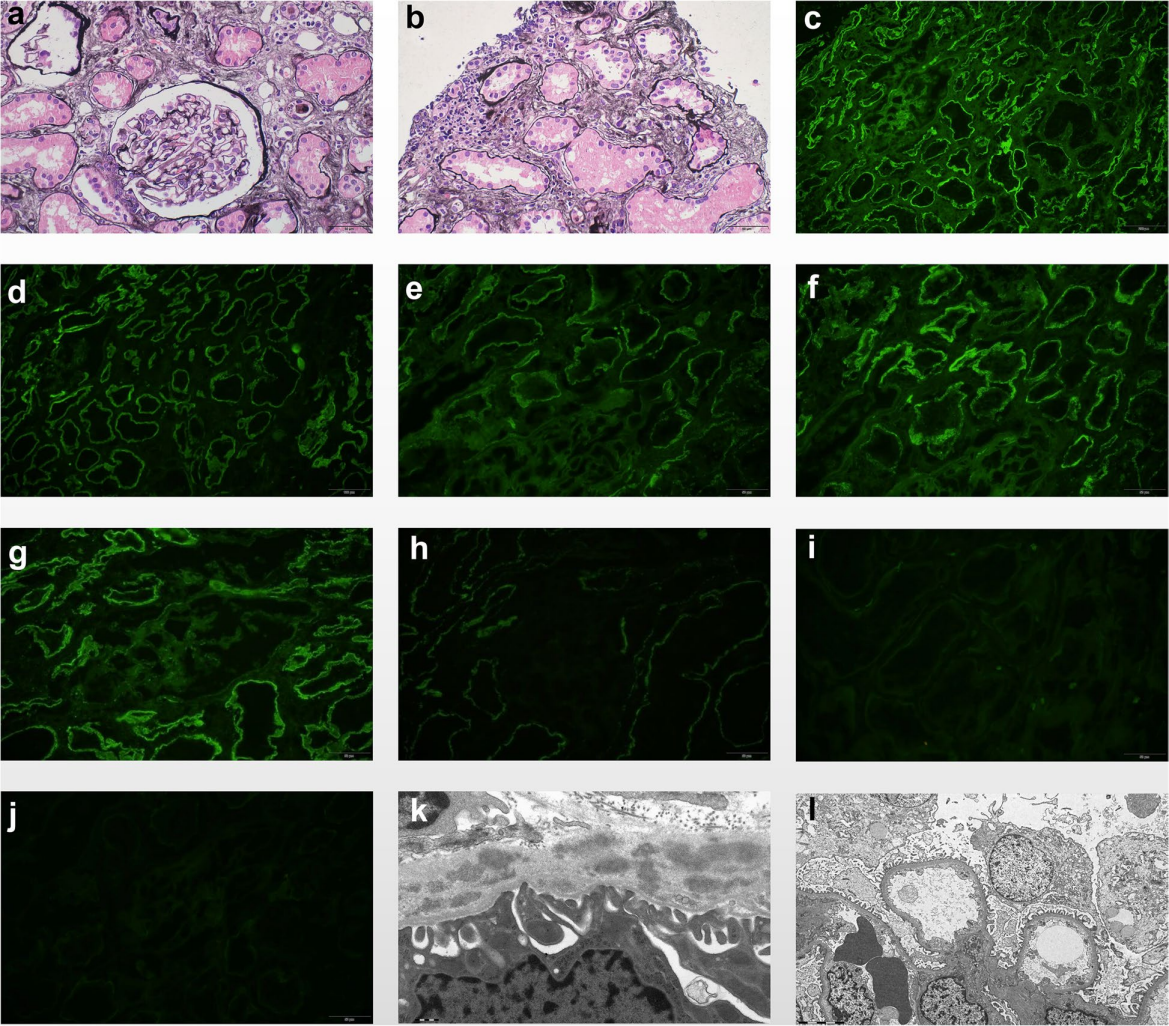
Histopathology of renal biopsy. **a**, **b** Mild-moderate tubular atrophy and interstitial fibrosis with patchy inflammatory cell infiltration in the interstitium was noted on PASM stain. Glomeruli look pretty normal (original magnification × 400). **c**-**f** Diffuse and granular TBM and focal Bowman’s capsule staining for IgG (3 +) (original magnification × 100), C3(2 +) (original magnification × 200), κ(2 +) (original magnification × 400), λ(3 +) (original magnification × 400) was shown on immunofluorescence. Only marginal staining was noted in mesangial area. **g** IgG subclass demonstrated IgG1(3 +) positivity, which is similar to IgG (original magnification × 400). **h** IgG4 (1 +) only deposited along TBM, while glomerulus is negative (original magnification × 400). **i**-**j** Both IgG2 and IgG3 were negative (original magnification × 400). **k**-**l** Electron microscopy showed numerous small granular or elongated electron dense deposits along both the proximal (original magnification × 25000) and distal tubular basement membrane (not shown). However, glomerular deposits were absent (original magnification × 5000)

A kidney biopsy was performed. On light microscopy, the specimen contained 2 cores with 25 glomeruli, 4 of which showed global sclerosis. There is no mesangial matrix increase or hypercellularity in glomeruli. Patchy interstitial fibrosis and inflammatory cells infiltration was noted in the interstitium. Mild-moderate tubular atrophy was observed (Fig. 1a-b). Several arteries exhibited mild intimal fibrosis. On immunofluorescence using a scale of 0–3 + , diffuse and granular staining for IgG (3 +) (Fig. 1c), C3 (2 +) (Fig. 1d), κ-light chain (2 +) (Fig. 1e) and λ-light chain (3 +) (Fig. 1f) along TBMs and a few Bowman capsules was seen. Only minimal staining was noted in a few mesangial areas. Additional IgG subclasses consisted of IgG1 (3 +) (Fig. 1g) and IgG4 (1 +) (Fig. 1h) in the TBMs. Of note, there was faint staining for IgG1 in Bowman capsules and segmental mesangial areas, while IgG4 was negative in glomeruli. IgG2 (Fig. 1i) and IgG3 (Fig. 1j) were both negative. Ultrastructurally, abundant granular or elongated electron dense deposits with a range of sizes were noted throughout the entire thickness of the proximal (Fig. 1k) and distal (not shown) tubular basement membranes. No obvious substructure was seen in the higher magnification. However, glomerular deposits were absent in two available glomeruli (Fig. 1l). Mild multilayering of peritubular capillary basement membranes (3–5 layers) were also observed.

**Fig. 2:**
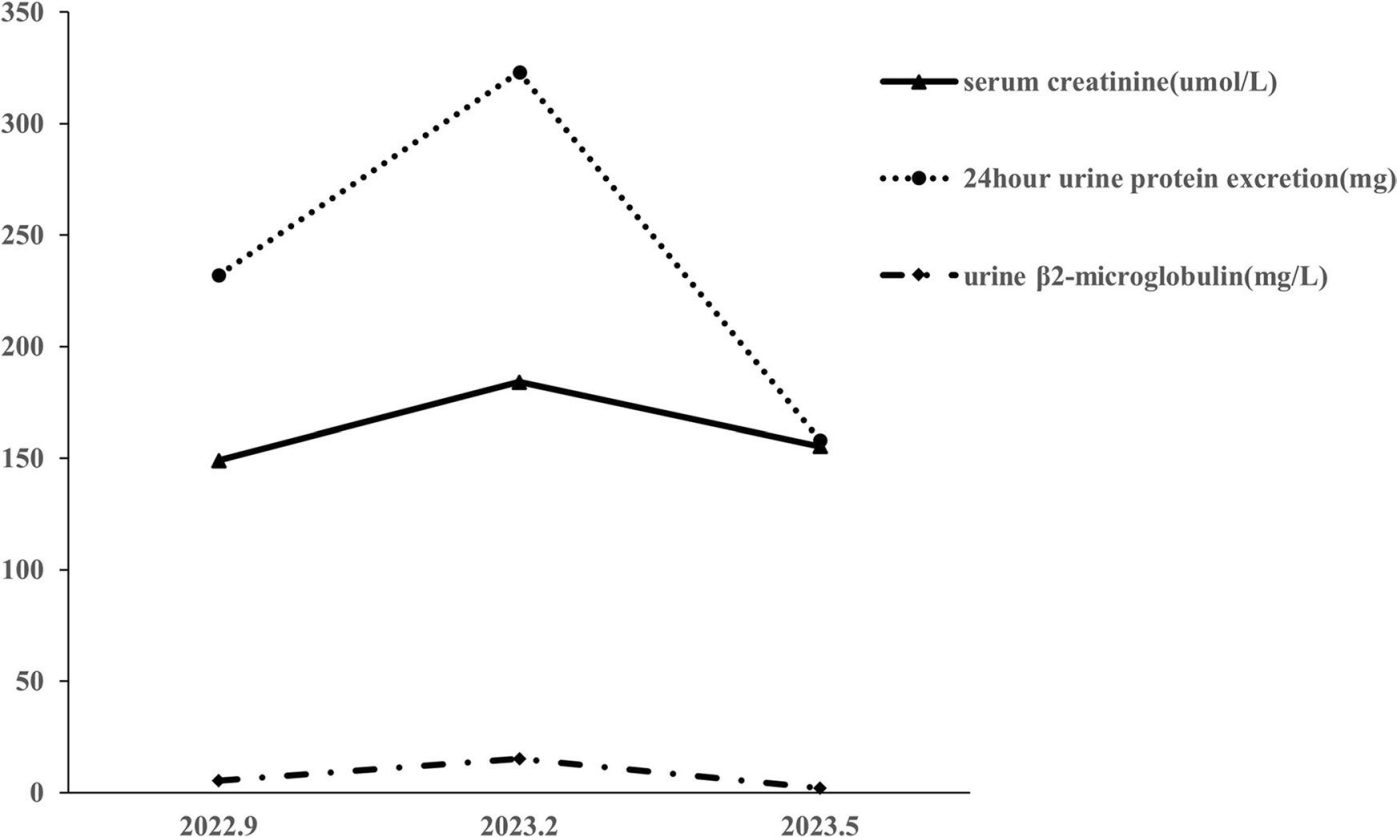
The clinical course. Changes of the serum creatinine, 24-h proteinuria and urine β_2_-microglobulin before and after treatment. Treatment of glucocorticoid initiated in the February 2023

The patient was treated with oral prednisone at 30 mg per day for one month, and then gradually tapered to 10 mg per day. After 12 weeks, his serum creatinine slightly decreased to 150 umol/L (Fig. 2).

## Discussion

Renal complications in HSCT recipients may be related to a combination of factors including chemotherapy, radiation, infection, immunosuppressive agents, ischemia, and cGVHD, and can involve glomerular, tubulointerstitial, and vascular structures [2–4]. In a kidney biopsy series of 20 patients with allogenic and autologous HSCT by Chang et al., thrombotic microangiopathy was the most frequent pathology identified, whereas membranous nephropathy (MN) was the most common form of immune complex-mediated glomerulonephritis [5, 6]. Besides glomeruli, extraglomerular immune complex deposits have also been demonstrated in the literature. Membranous changes with a lumpy deposition of IgG and complement along the TBM in a bone marrow transplant recipient was first described by Ohsawa [7]. Kudose reported three MN patients with granular TBM deposits following allogeneic HSCT, and two of these were NELL-1 positive in glomeruli. Of note, NELL-1 was negative along TBM [8]. A recent series of 5 cases from Nasr et al. revealed global subepithelial and extensive proximal TBM immune complex deposits [1]. IgG subtypes staining in 2 cases showed sole or dominant IgG4 subclass positivity. PLA2R was positive in glomeruli in 1 of 3 patients tested, but also negative along TBM. The latest study identified a novel protein, protocadherin FAT1 is related to most cases of HSCT-associated MN [9]. Interestingly, 3 cases showed TBM deposits that were positive for IgG. But mass spectrometry of the TBM deposits was still negative for FAT1. It is proposed that membranous nephropathy with extensive TBM deposits is a distinctive clinicopathologic lesion associated with allogeneic HSCT [1]. Pathogenesis likely involves cGVHD-driven antibodies against glomerular and TBM components. However, the target antigen of the TBM deposits does not appear to be the same as that in glomeruli. Furthermore, Satoskar et al. demonstrated that the IgG subclass composition of the TBM and vascular wall immune complex deposits often differ from the glomeruli in a majority of lupus nephritis biopsies, which may point towards different pathways in the formation of glomerular and extraglomerular immune complexes [10]. To date, there have been no reports of predominant granular TBM immune-complex deposits of IgG, C3, κ, and λ with faint glomeruli deposition following allogeneic HSCT. Mild multilayering of peritubular capillary basement membranes in our patient indicated the possibility of cGVHD. We suspected that the generation of unique antibodies against the TBM components or cross-reacting antibodies is the likely etiology. IgG subclass distribution was discrepant between glomerular and TBM deposits in our patient and raises the possibility that different mechanisms may be playing a role in immune complexes formation and subsequent tissue injury. In addition, IgG1 dominance in our patient may facilitate fixation of the complement system and favored an underlying autoimmune disease or other secondary kidney disease [11, 12]. Further studies investigating the TBM targets are needed to understand the pathogenesis of our case.

TBM deposition is also found in a variety of cases in native kidney biopsy. It is classified as polyclonal and monoclonal pattern according to the heavy and light chain staining, and also is divided into two categories based on the deposition morphology (linear or granular). Our case showed electron dense deposits along the TBM of both proximal and distal tubules, contrary to anti-brush border autoantibody tubulointerstitial nephritis in which they are only limited in proximal tubules and a few capillary walls [13, 14]. Anti-TBM disease manifests as linear deposition along TBM with absence of immune deposits [15], which is also different from our case. Autoantibodies known to cause IgG deposition in TBM, such as in lupus, Sjogren syndrome, IgG4-related disease were all negative. The data of virus index, inflammatory biomarkers, and complement levels were in the normal range, and the patient also did not use calcineurin inhibitor or other nephrotoxic drugs at the time of biopsy. Therefore, TBM deposition in our patient has less correlation with some common autoimmune diseases, and drug induced tubulointerstitial nephritis. DNAJB9-positive tubulointerstitial-predominant fibrillary nephritis was reported recently [16], which showed focal linear staining with IgG and fibrillar deposits along TBMs. No fibrils were noted the TBMs of our case. In the setting of kidney transplantation, polyomavirus infection with granular TBM deposition has been observed in renal transplant recipients [17]. However, negative virus detection in our patient ruled out the possibility. Anti-glomerular and anti-TBM nephritis was also a rare manifestation for renal allograft recipient with Alport syndrome [18]. Compared to the linear pattern in the former, our patient manifested with a granular pattern along TBM. Moreover, monoclonal IgG TBM immune deposits with progression of IF/TA were observed in some renal allografts [19], which was different from polyclonal staining in our patient.

Our study has several limitations. First, serum was not collected at the time of renal biopsy, so we cannot determine whether antibodies against TBM components exist or not. Second, there is no definite evidence of cGVHD in our case.

## Conclusion

In summary, we reported a unique case of diffuse granular staining with polytypic IgG deposited along the TBM following allogeneic HSCT. Generation of unique antibodies against the TBM components or cross-reacting antibodies is the likely etiology, the identity of which remains for further research.

## Data Availability

The datasets used and analyzed during the current study are available from the corresponding authors on reasonable request.

## Abbreviations

TBM: Tubular basement membrane
HSCT: Hematopoietic stem cell transplantation
cGVHD: Chronic graft-vs-host disease
MN: Membranous nephropathy
